# Destruction mechanisms of ozone over SARS-CoV-2

**DOI:** 10.1038/s41598-021-97860-w

**Published:** 2021-09-22

**Authors:** Angila Ataei-Pirkooh, Ali Alavi, Mehran Kianirad, Kowsar Bagherzadeh, Alireza Ghasempour, Omid Pourdakan, Reza Adl, Seyed Jalal Kiani, Mehdi Mirzaei, Bita Mehravi

**Affiliations:** 1 Department of Virology, School of Medicine, Iran University of Medical Sciences, Tehran, Iran; 2 Research Center for Clinical Virology, Tehran University of Medical Sciences, Tehran, Iran; 3 Department of Chemistry, Science and Research Branch, Islamic Azad University, Tehran, Iran; 4 Department of Biotechnology, Iranian Research Organization for Science and Technology, Tehran, Iran; 5 Nano Fanavari Kian Gostar Company, Technologies Incubator Center, Iran University of Medical Sciences, Tehran, Iran; 6 Stem Cell and Regenerative Medicine Research Center, Iran University of Medical, Tehran, Iran; 7 Eye Research Center, The Five Senses Institute, Rassoul Akram Hospital, Iran University of Medical Sciences, Tehran, Iran; 8 Department of Medical Nanotechnology, Faculty of Advanced Technologies in Medicine, Iran University of Medical Sciences, 1449614535 Tehran, Iran; 9 Finetech in Medicine Research Center, Iran University of Medical, Tehran, Iran; 10 Department of Chemistry, Faculty of Sciences, Shahid Beheshti University, Tehran, Iran; 11 Iran Ministry of Health and Medical Education, Deputy Ministry for Education, Tehran, Iran

**Keywords:** Infectious-disease diagnostics, Antiviral agents

## Abstract

In this pandemic SARS-CoV-2 crisis, any attempt to contain and eliminate the virus will also stop its spread and consequently decrease the risk of severe illness and death. While ozone treatment has been suggested as an effective disinfection process, no precise mechanism of action has been previously reported. This study aimed to further investigate the effect of ozone treatment on SARS-CoV-2. Therefore, virus collected from nasopharyngeal and oropharyngeal swab and sputum samples from symptomatic patients was exposed to ozone for different exposure times. The virus morphology and structure were monitored and analyzed through Atomic Force Microscopy (AFM), Transmission Electron Microscopy (TEM), Atomic Absorption Spectroscopy (AAS), and ATR-FTIR. The obtained results showed that ozone treatment not only unsettles the virus morphology but also alters the virus proteins’ structure and conformation through amino acid disturbance and Zn ion release from the virus non-structural proteins. These results could provide a clearer pathway for virus elimination and therapeutics preparation.

## Introduction

SARS-CoV-2 is the causative agent of the COVID-19 disease, which has a significant mortality rate^1,2^. The spread of virus particles over surfaces or objects in the immediate environment around an infected person is considered a major method of virus transmission. Therefore, a rapid and effective disinfection process without any particular environmental side effects would be benifical^3^. Many useful disinfecting methods, including UV radiation, chlorine-based disinfectants, and hydrogen peroxidase vapor, have been reported, but most of these have limitations, including the inability to access all surfaces as well as long time requirements, which can lead to potential skin disease in UV-based techniques. Among the reported methods, the ozone treatment seems very promising^4–7^. Ozone is the most powerful oxidant found in nature^6–8^, and due to its instability-it decomposes by itself- the ozonization process only needs a short exposure time, whereby there are no harmful residuals that need to be removed after ozonization processes^8,9^. The potential usefulness and benefit of the ozonization disinfecting process (ODP) have already been reported^9–12^. Although the beneficial effects of Ozone treatment against many viruses have been reported since the 1800s, the inactivation mechanism of action is still relatively unknown^13^. Some studies have reported the effect of ozone on cysteines in the surface proteins of coronaviruses, in which ozone and other oxidants can inhibit virus entry into host cells^14^.

The aim of this study was to investigate the effect of ozone on this virus and to find the mechanisms of its effect so that ozone can be used as a disinfectant in different places in current epidemic conditions. Also, possible mechanisms of the effect of ozone on viruses have been expressed in a scattered manner in previous studies, so this study also explains several mechanisms together. The obtained results showed that ozone eliminates coronavirus via three mechanisms, including conversion of cysteine to cystine, disturbance of the viral envelope formation, and the release of Zn^+2^ from the virus proteins’ structures.

## Results and discussion

**Figure 1:**
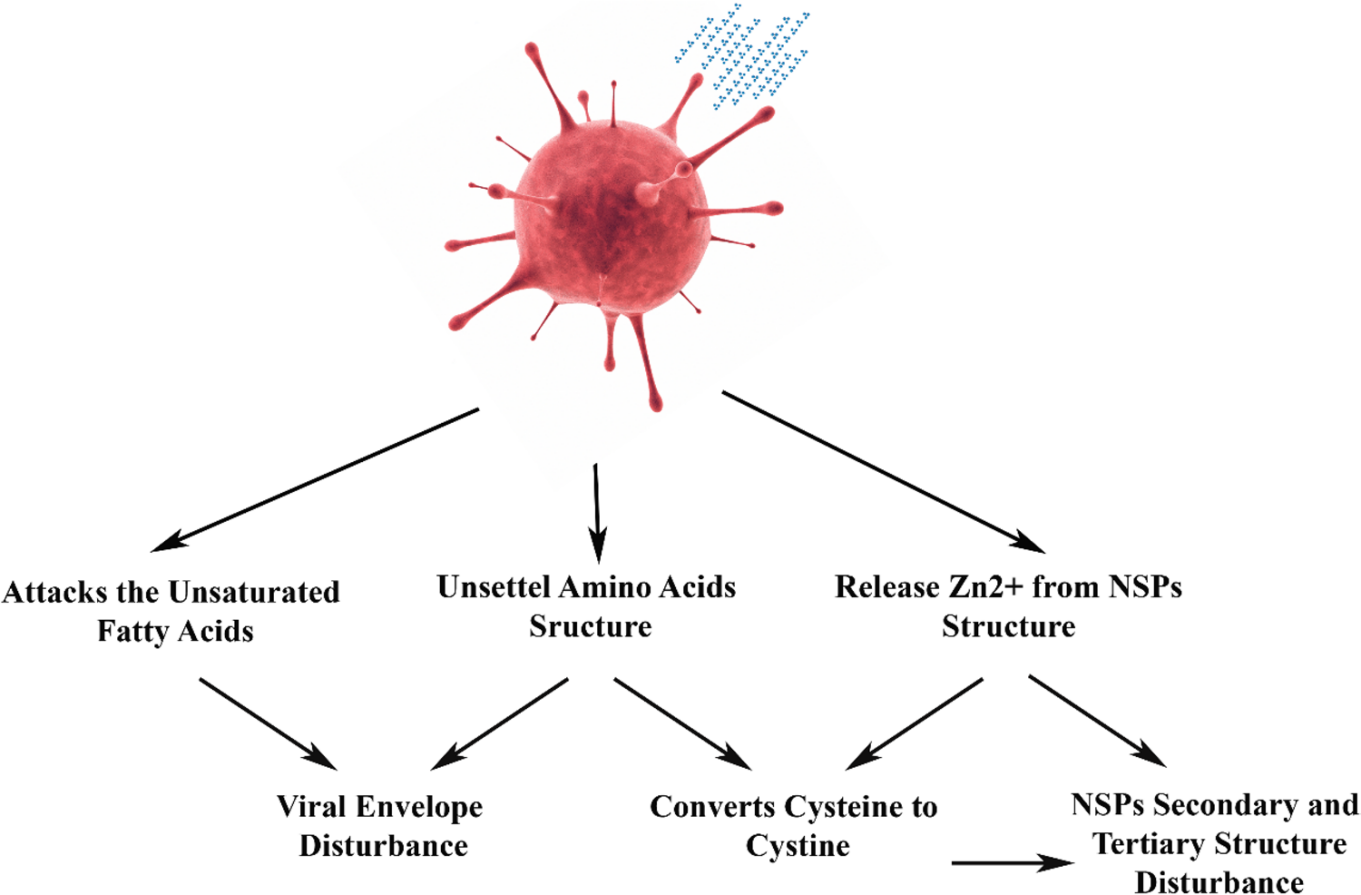
Schematic diagram of ozone destruction mechanism over SARS-CoV-2.

**Figure 2:**
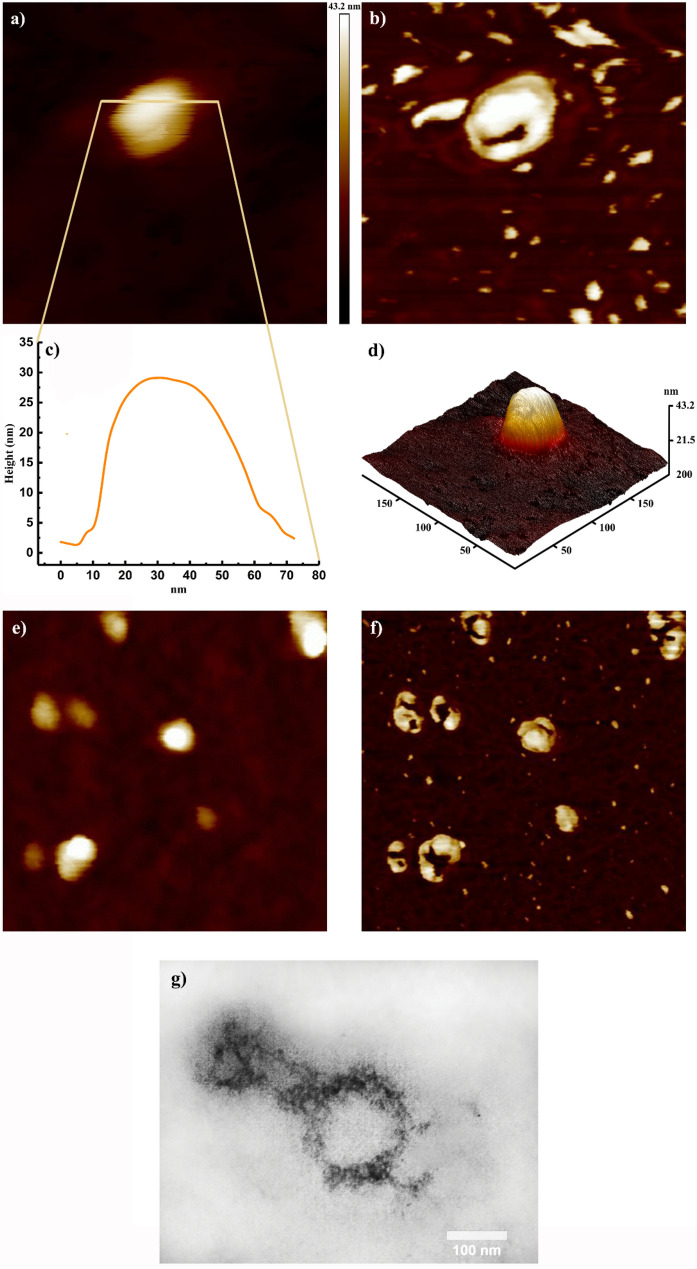
Topographic AFM image of the ozone treated SARS-CoV-2 particle on Glu-UD-Si(100) surface: (**a**) 2D image of a single virus particle (200 × 200 nm), (**b**) AFM phase image of a single virus particle with the virus debris (200 × 200 nm), (**c**) The corresponding height profile of the virus, (**d**) 3D image of the same virus, (**e**) AFM height image of the virus particles (500 × 500 nm), (**f**) AFM phase image (500 × 500 nm), and (**g**) Transmission electron microscopy imaging of ozone treated SARS-CoV-2. A negative-stained virus particle shows features of coronaviruses (60,000 ×).

The ozonization process effect over SARS-CoV-2 was thoroughly studied in the present study (Fig. 1). In this regard, the ozone-treated virus was first covalently bonded to the glutamine modified surface of silicon “(SARS-CoV-2)-GLU-UD-Si(100)” and then analyzed using AFM and ATR-FTIR techniques; further analyzes were performed using AAS and transmission electron microscopy. Figure 2a shows the 2D topographic AFM image of a single ozone-treated virus particle and Fig. 2b,c show an AFM phase image and the corresponding height profile of the same particle, respectively. Also, a 3D image of this particle is shown in Fig. 2d. Figure 2e,f show the height and phase images of some ozone-treated virus particles in a microscopic field. Also, Transmission electron microscopy showed the round shape of a damaged virus particle with an average size of 80–100 nm (Fig. 2g). The exposure time in Fig. 2 is 2 min.

### Ozone disturbs the viral envelope

Coronaviruses, especially the SARS-CoV-2, are rich in cysteine, and their residues must be intact for viral activity, mainly through zinc ion participation^14,15^. It is known that conserved cysteine residues are important in virus stability and the loss of more than one cysteine affects the formation of the viral envelope^14^. It seems that upon exposure to ozone, the viral surface of SARS-CoV-2 became more heterogenous than the native virus (Fig. 2b). Desorption of proteins occurs from the boundaries of the membrane patches, gradually leading to the release of proteins. As it is observed in both lateral phase images (Fig. 2b,f), many debris were liberated and dispersed near the virion itself, which might be attributed to the breakdown of the viral envelope or release of proteins caused by ozone treatment. Kuznetsov et al. studied the effect of detergents on HIV using the AFM technique and observed similar viral fragments, proteins, and viral debris^16^. Further, ozone attacks the unsaturated fatty acids in the membrane with the possible consequence of lipid peroxidation^9^, which is observed by increased malondialdehyde, one of the aldehydic products of lipid peroxidation^17^.

ATR-FTIR analysis (Supplementary Information, Figure S1) shows a blue shift in ʋ_as_(CH_2_), from 2924.42 to 2919.68 cm^−1^. It was reported that two spectra are observed at 2924 and 2920 cm^−1^ for single planar phospholipid bilayers and dry phospholipid^18^. Accordingly, the shift can be attributed to an ozone attack on the virus phospholipid bilayers in the viral envelope that disturbs the envelope’s proper structure. Additionally, the pH decreases in the viral suspension with increasing exposure time as a result of carboxylic acid formation (the wide peak from 3200 to 3400 cm^−1^), which further proves the presence of the OH group of carboxylic acid on the surface (Supplementary Information, Table S1). Another slight blue shift is evident in the IR-spectra (the right spectra) from 1656.56 to 1654.22 cm^−1^ in the amide I region. The intensity of these modes shows that the phospholipid layers are cramped, packed, and torn.

### Ozone converts cysteine to cystine

Ozone exposure results in protein secondary and tertiary structure destruction^9^. It has been observed that ozone attacks the amino acids directly, particularly cysteine, methionine, tryptophan, tyrosine, and histidine^9^. Upon exposure to ozone, cysteine is oxidized as the first step to cystine, but further oxidation by ozone converts cysteine to cystic acid^8,12^. Due to the important role of conserved cysteine residues, the conversion of cysteine to cystine was monitored over 20 min of exposure to ozone (Supplementary Information, Figure S2). As observed in Supplementary Information, Figure S3, the intensity of the SH bond decreased dramatically, probably because of cysteine (disulfide bond) formation.

**Figure 3:**
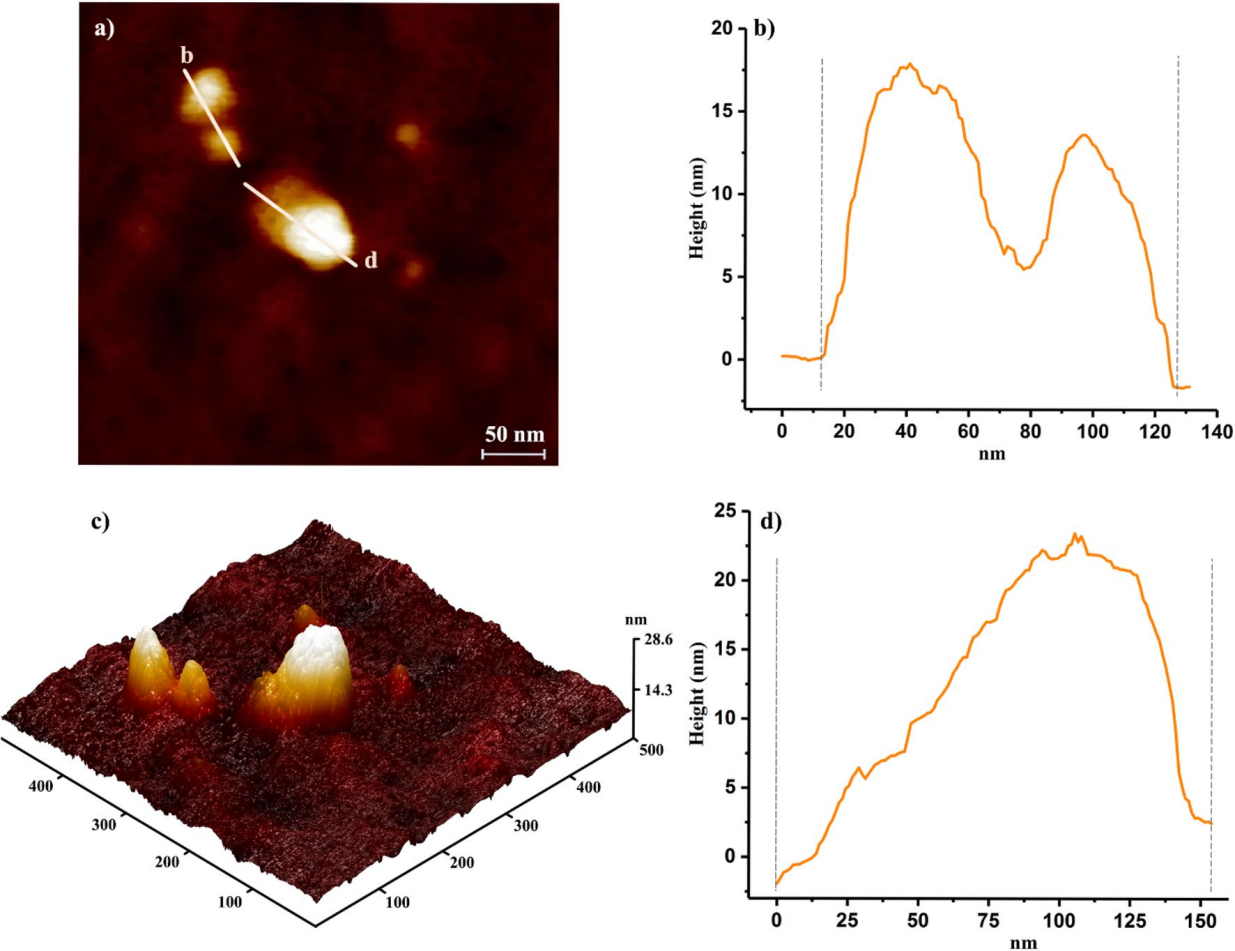
AFM images of the virus aggregation at 2 min ozone exposure time; (**a**) 2D image of aggregated virus particles. (**b**) and (**d**) The corresponding height profiles of the aggregated virus particles reflecting AFM image of (**a**) and (**b**). (**c**) 3D image of the same virus particles.

**Figure 4:**
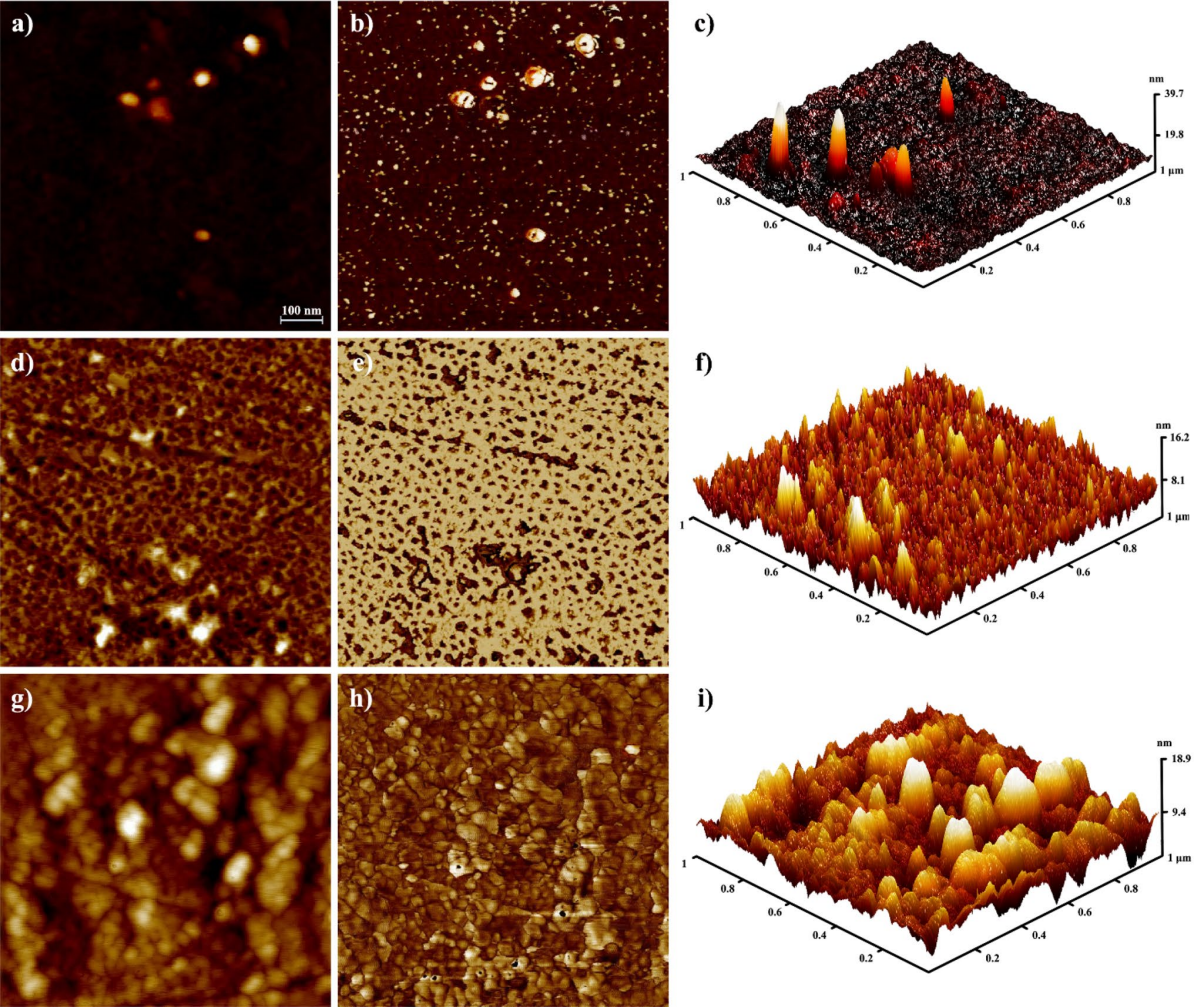
AFM images showing the effect of ozone on the virus at different times (2, 4, and 6 min) in (**a**), (**b**), and (**c**), respectively. After 2 min of exposure to ozone (2D, lateral, and 3D views), (**d**), (**e**), and (**f**), respectively. After 4 min of exposure to ozone (2D, lateral, and 3D views), (**g**), (**h**), and (**i**), respectively. After 6 min of exposure to ozone (2D, lateral, and 3D views).

The viral suspension samples were also exposed to ozone and were then evaluated by transmission electron microscopy and AFM techniques. The TEM image, Supplementary Information, Figure S4, shows two connected virus particles; it seems SH groups from adjacent viruses are linked to form disulfide bonds after 2 min of ozone exposure. After 2 min, they bind together due to the formation of a disulfide bond as it is shown in Fig. 3a–d, and after 4 and 6 min of exposure time, the viruses are totally damaged, and the remaining components are aggregated, as observed in Fig. 4. The effect of ozone on the morphology of the virus at different exposure times (2, 4, and 6 min) has also been investigated, as shown in Fig. 4 (will be discussed) later. Additionally, ATR–FTIR was used to indicate protein denaturation^19^. The IR-spectra was taken after every modification step and are shown in Supplementary Information, Figure S3. It is observed that cysteine residue SH groups readily oxide to disulfide upon ozone exposure.

### Ozone releases Zn^+2^ from the virus proteins

As it is already mentioned, the virus cysteine residues need to be intact in the virus protein structures, mainly through zinc ion participation, to be able to play their crucial role in the viral activity^14,15^. Zinc ions participate in many different cellular processes, and zinc-cysteine finger motifs have been proven to play a crucial role in proper protein-folding, catalysis, regulation of various enzymes, transcription factors, and other activities still under study. A group of these finger domains that stabilizes protein structures can interact with other biomolecules, such as RNA and DNA.As previously reported, exposure to oxidative conditions results in Zn^2+^ ion removal from proteins’ zinc-cysteine finger motifs and disulfide bond formation between the cysteine residues, which consequently denaturize proteins’ structure and alter their solubility^20,21^. Anja Seybert et al. revealed that during protein refolding in the denaturation-renaturation process, Zn^2+^ is essential for the rescue of the enzymatic activities of coronavirus helicases^22^. Atomic absorption spectroscopy was applied to measure the concentration of zinc in the supernatant after being exposed to ozone for 2 min. The zinc concentration was found to be approximately one part per million. It was observed that the attachment of the zinc-cysteine complex to the silicon surface is lost upon the release of zinc from cysteine moiety as a result of ozone exposure (Supplementary Information, Figure S3).

Zinc ions participate in many different cellular processes, and zinc-cysteine finger domains have been proven to have a crucial role in the proper folding, catalysis, and regulations of various enzymes and transcription factors^23,24^. Zinc/sulfur enzymes frequently lose their catalytic activity after the oxidative release of the catalytic metal ion^25^. Coronaviruses employ a set of non-structural proteins (nsp) produced as cleavage products of the ORF1a and ORF1ab (viral polyproteins that assemble to assist viral replication and transcription)^24^. Zinc finger motifs are observed in SARS-CoV-2 non-structural proteins, especially nsp3 (PDBID: 6W9C), nsp10 (PDBID: 6WKQ), and nsp12 (PDBID: 7BTF)^26–28^.

**Figure 5:**
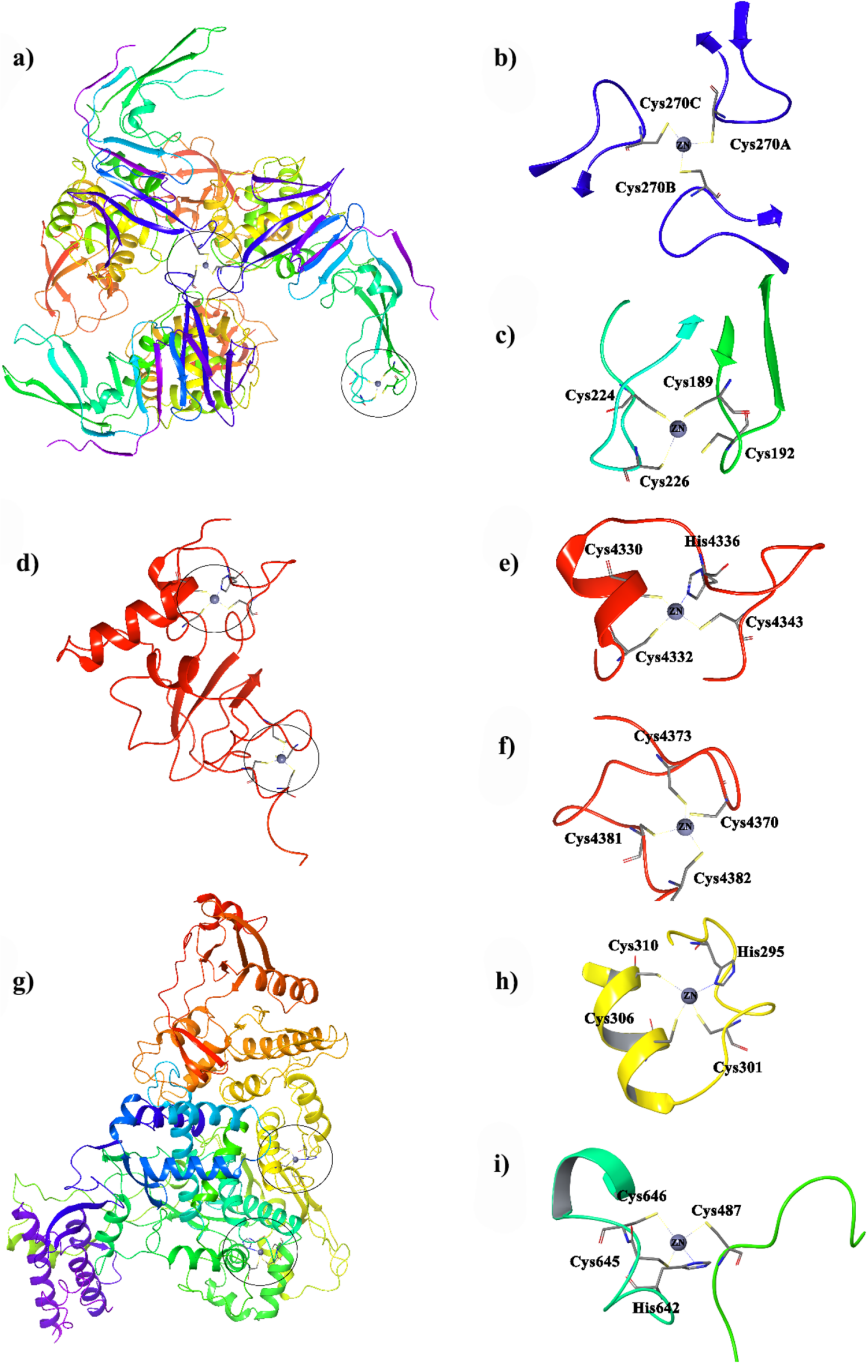
(**a**) SARS-COV-2 nsp3 (papain-like protease; PLpro, PDB ID: 6W9C) and the corresponding zinc motifs in (**b**)**,** (**c**), and (**d**). SARS-COV-2 nsp10 (PDB ID: 6WKQ) and the corresponding zinc motifs (**e**), (**f**), and (**g**). SARS-COV-2 nsp12 (RNA-dependent RNS polymerase; RdRp, PDB ID: 7BTF) and the corresponding zinc motifs (**h**) and (**i**).

Two types of zinc finger motifs structurally stabilize nsp3 for interaction with other proteins and biomolecules (Fig. 5a). According to Fig. 5b, four cysteine residues, provided by two β-hairpins, chelate Zn^2+^ ion in the zinc ribbon of the protein finger domains (Fig. 5c). Exposure to oxidative conditions results in Zn^2+^ removal from the finger along with disulfide bond formation between the cysteine residues and consequently decreases enzymatic activity or inactivates the protein^20,21^. Zn^2+^ ion chelation of cysteine residues at the protein interface modulates a supermolecular assembly that subsequently enables enzymatic activity. According to Fig. 5b,a cysteine residue (Cys270) from each domain chelate Zn^2+^ ion for a proper trimer formation. These cysteine residues are sensitive to redox conditions, and upon exposure to oxidative agents readily form disulfide bonds and release a Zn^2+^ ion that results in the formation of free monomers and consequently disrupts the enzyme activity^29^.

Nsp10 has a considerable role in the virus replication/transcription machinery. It has been shown that the nsp14–nsp10 complex is involved in RNA viral proofreading^30–32^. Moreover, nsp10-nsp16 interactions activate nsp16-mediated 2′-O-MTase activity, which avoids or delays viral RNA recognition by host innate immune sensors highlights.

Accordingly, nsp10 is considered a great target to develop broad-spectrum inhibitors for repressing coronaviruses activity^33^. Nsp10 includes two zinc finger motifs (Fig. 5d). The first (Fig. 5e, with three cysteines and a histidine residue) belongs to a new Zn finger family and is proposed to possibly have an RNA-binding function (one chelated), and the other one (Fig. 5f, chelated with four cysteine residues) has a typical Zn finger motif required for the structural integrity of the C-terminal half of the structure as well as its oligomerization and is highly conserved in all coronaviruses^30,34^. Studies have shown that mutation in Zn coordinated residues abrogate nsp10 interactions with nsp14 and nsp16, probably due to the protein misfolding^35^. It should also be mentioned that SARS Coronavirus nsp14 includes three Zn finger motifs from which the one in the proximity of the active site plays a role in the catalysis, and the other two are responsible for the enzyme structure stability^25,30^.

Nsp12 (RNA- dependent RNA polymerase; RdRp) contains two zinc fingers (Fig. 5g–i), each coordinated to three cysteines and a histidine residue^36^. Computational studies suggest that the zinc motifs are a part of a conserved mechanism of RdRp switching, which changes RdRp domain orientations to stabilize the protein structure in different phases of its catalytic activity^37^. Ultimately, exposure to oxidative agents, like ozone, would result in disulfide formation and loss of Zn^2+^ from the binding site of the virus’ key nsps that inhibit the enzyme activity or alter its proper function.

### The effect of ozone on the virus depends on time

The effect of ozone exposure on the virus was monitored at different times (Fig. 4). At the exposure time of 2 min, the viruses are damaged and destroyed. As can be seen in Fig. 4c, the height of the particles in the AFM image appears to be higher due to the formation of empty particles. It seems the viruses that have lost their genomes appear narrower in the 3D image because of the finite size of the AFM tip. Increasing the exposure time results in the formation of new crystals, probably from the remnants of the damaged virus particles.

## Conclusions

In this study, the effect of ozone on SARS-CoV-2 virus and its was investigated. AFM images showed significant heterogeneity on the surface of viruses after ozone treatment. The results of atomic absorption spectroscopy and transmission electron microscopy showed that ozone breaks down virus proteins through three mechanisms; it destroys the virus envelope, converts cysteine to cystine through disulfide bond formation between adjacent virus particles, and finally release Zn ions from the virus protein structures which eventually destroy the structure of the proteins and alter their proper activity. In addition, increasing the time of ozone exposure leads to the formation of new crystals, which may be damaged by the remnants of virus particles. The obtained results would definitely expand our knowledge to develop virus elimination techniques as well as potential therapeutics and treatment mechanisms employing oxidizing agents like ozone.

## Materials

10-Undecenoic acid (98%), acetic acid (99%), N-hydroxysuccinimide (NHS) (99%), N,N′-Dicyclohexylcarbodiimide (DCC) (99%), Pyridine (%99), 4-(Dimethylamino)pyridine (DMAP) (99%), and Zinc acetate were purchased from Merck. L(+)-Glutamine (99%), N,N-Dimethylformamide (DMF) (99.8%), chloroform (99%), 1-heptene (97%), L(+)-Glutamine (Aldrich, 99%) and Cysteine (98%) were obtained from Aldrich and used as received. Dichloromethane (DCM) (99%) was from Caledon. The sodium and potassium chloride, Na2HPO4 and KH2PO4, had a purity of ≥ 99% (all from Merck and Aldrich). Phosphate buffered saline (PBS, 10 mM, pH 7.4) was prepared from a solution of Na2HPO4 (1.41 g/L), KH2PO4 (0.27 g/L), NaCl (8.01 g/L) and KCl (0.20 g/L) in deionized (DI) water. The pH was adjusted either by ultra-pure NaOH or HCl solutions before adjusting to the final volume. Hydrofluoric acid (49%), sulfuric acid (96%), hydrogen peroxide (30%), and ammonium fluoride (40%) were all VLSI grade chemicals purchased from Aldrich, Merck, and GEM Microelectronic Materials (Arizona, USA). MilliQ ultrapure water (18.2 MΩ cm, 1 μg/L total oxidizable carbon) was used for solvent treatment, cleaning, and solution preparations. A silicon wafer (single side polished Si(100), n-type, phosphorous doped, 1–10 Ω cm resistivity, 0.2 miscut toward the (110) direction, and 475 ± 25 μm thickness) was obtained from Virginia Semiconductor and used for the AFM studies. Also, a Si(100) wafer (p-type, B-doped, dabble side polished, 4–15 Ω.cm, 600–650 μm thickness from UniSil Corporation) cut and polished (with diamond powders graduated from 10 to 0.01 µm) into a trapezoid parallelogram shape (10 × 27 mm, 45° bevels) with ~ 43 reflections was used for the ATR-FTIR analysis.

## Methods

### Virus collection, detection, and preparation

Specimen collection was carried out according to CDC guidelines for handling and testing clinical specimens for SARS-CoV-2^38^. Patients spike samples were considered for the study if they had given written informed consent and had clinical evidence of COVID-19 confirmed by a PCR test. The work was conducted in compliance with principles of the Declaration of Helsinki and is also approved by the Iran University of Medical Sciences Ethics Committee with an ethics code of IR. IUMS.REC.1399.329.

Nasopharyngeal and oropharyngeal swab and sputum samples were collected from symptomatic patients to detect SARS-CoV-2 by real-time reverse transcriptase assay. RNA extraction (from 500 µl of each sample) was performed using the QIAamp DSP Virus Kit (Qiagen GmbH, Hilden, Germany) according to the manufacturer’s protocols. Briefly, the Qiagen protease was added to the lysis tube and mixed with 500 µl of the sample. Subsequently, the carrier RNA-containing lysis buffer was added to the tube and mixed by pulse-vortexing. The tube was incubated at 56 °C for 15 min. Ethanol was added to the tube and incubated for 5 min at room temperature. The lysate was applied into columns connected to a vacuum system. After performing the washing steps, the tube was centrifuged at full speed for 1 min to completely dry the membrane. RNA was eluted from the membrane in 50 µl of the elution buffer and stored at − 80 °C for further analysis.

A qualitative multiplex real-time PCR assay was recruited to detect SARS-CoV-2 using a Novel Coronavirus (2019-nCoV) Nucleic Acid Diagnostic Kit (Sansure Biotech, Changsha, China), according to the manufacturer’s instructions. Briefly, a reaction mastermix containing the PCR mix (primers, probes, dNTPs, MgCl2, RNasin, and buffer) and the enzyme mix (RT and Taq) was prepared for all samples. Twenty microliters of the sample were added to each tube. Positive control, negative control, and no-template control (NTC) were also included in the test. The assay was performed on a Rotor-Gene Q qPCR machine (Qiagen, Hilden, Germany) with the following cycle parameters: 1 cycle of reverse transcription at 50 °C for 30 min, 1 cycle of cDNA predenaturation at 95 °C for 1 min, 45 cycles of denaturation at 95 °C for 15 s, and an annealing-extension at 60 °C for 30 s (fluorescence collection). The results were considered positive if there were typical S-shape amplification curves detected at Ct ≤ 40.

Forty samples, which had higher viral loads, were pooled and then divided into different containers and exposed to ozone for different times (from 2 to 6 min). Ozone was produced through an ozone generator (ozone solution model) at a concentration of 5 mg/h.

### Surface preparation for the virus attachment and analysis

#### Surface preparation and the virus attachment

The silicon wafer and ATR crystal were prepared according to a study by Alavi et al.^39^. Trace amounts of organic containment were removed using Piranha solution^40^. Afterward, the silicon surfaces were placed in a PTFE vial containing 15 ml of NH4F solution (VLSA grade 40%) and purged with argon bobble for 30 min. The obtained H-Si(100) surface was then washed with deoxygenated MilliQ water, dried, and immediately placed into a quartz Schlenk tube containing 20 ml of deoxygenated neat undecanoic acid (UD), then purged with argon for 30 min, and finally, exposed to UV illumination (365 nm, Black-Ray B-100 AP Lamp, 100 W) 4 h^41^. The UD-Si(100) surface was then functionalized with a freshly prepared solution of mixed NHS (0.1 M) and DCC (0.4 M) in a deaerated anhydrous DMF (3 mL) for 2 h while being argon bobbled at room temperature (25 ± 1)^42,43^. The obtained NHS-UD-Si(100) monolayers were rinsed and sonicated with dried DMF and DCM, then dried under argon stream and placed in 5 ml of a saturated solution of glutamine in deoxygenated DMF containing a small amount of anhydrous pyridine (0.04 to 0.06 g) as a catalyst while being purged with argon at room temperature (25 ± 1) for 2 h to give GLU-UD-Si(100).

PBS rinsed GLU-UD-Si(100) was dipped into a vial contained freshly ozone-treated viruses while being purged with argon for the desired reaction times. (SARS-CoV-2)-GLU-UD-Si(100) wafers or ATR- crystals were then spray rinsed with a PBS solution for 5 s, rinsed again with MilliQ water, and finally dried under an argon stream. The prepared surfaces were sent for ATR-FTIR, AFM, and water contact angle surface characterization immediately after each surface functionalization step.

#### Monolayer formations for Zn characterisation

Zn(OAc)_2_-Cys-APTES-SiO_x_ Si(100) functionalized surfaces, SiO_x_(100), and the Piranha cleaned wafer or ATR crystal were placed into a vial containing 10% APTES in Toluene for 24 h, then the silicon ATR crystal was removed and cleaned with copies amount of DCM, Methanol, and chloroform and dried under a pure argon stream. The dried APTES-SiO_x_ Si(100) ATR crystal was then placed in a vial containing 2 ml of DMF, DCC 0.04 g, L-cysteine 0.05 g, DMAP 0.001 g, and bubbled argon for 4 h at 100 °C. After the reaction was completed, the Cys-APTES-SiO_x_ Si(100) functionalized ATR crystal was removed and washed with lots of solvent like DCM, water, and methanol and sonicated in DCM for 10 s. Then the obtained ATR crystal was removed, dried with Argon gas, and placed in a solution of Zn(OAc)_2_ (0.1 g /2 ml) for 10 min at room temperature (25 ± 1). The resulting Zn(OAc)_2_-Cys-APTES-SiO_x_ Si(100) was washed with methanol and dried before ozone treatment.

#### Wafer preparation to investigate the effect of ozone on cysteine

0.3 g of cysteine was dissolved in 2 ml of Milli-Q water and was sparged with ozone for 2–20 min. After weight precipitation appeared, the sample was filtered and dried and the correspondence spectrum was graphed by diamond ATR.

### Characterization techniques

#### Atomic force microscopy (AFM)

AFM characterizations were performed in air using a DPN 5000 system from NanoInk in non-contact mode. The AFM probes were PEN-001-05, spring constant 0.1 (N/m), and PEN-0005-01, spring constant (40 N/m), 300 kHz (NanoInk), which were utilized for contact and non-contact AFM mode, respectively. Silicon tips (nominal tip radius ≤ 10 nm) were used for high-resolution imaging, spring constant ≈ 40 N/m, resonance frequency ≈ 300 kHz. AFM Imaging was performed in the attractive force regime, in which the tip is in very weak interaction with the surface (or low drive amplitude).

#### Transmission electron microscopy (TEM)

In the negative staining process, a drop of the viral suspension was placed immediately on the carbon-coated copper grid and left for 3–5 min. Next, the grid was drained with filter paper and negatively stained with 2% Uranyl acetate. Observations were made on a Zeiss LEO 906 electron microscope at 100 kV.

#### Atomic absorption spectroscopy (AAS)

Determination of zinc (after centrifugation in the supernatant) was carried out by atomic absorption spectroscopy, using a GF-AAS instrument (3.02 GBC Savant AA, Australia) and a hollow cathode lamp PerkinElmer (324.75 nm). The measurements were made employing an air acetylene flame with a spectral groove width of 0.5 nm. The solutions for the construction of the calibration curve were made from a standard solution Merck KGaA brand copper standard (1000 ppm). Viral transport media was used as a blank in this procedure.

#### ATR-FTIR measurement

ATR-FTIR measurements were performed using a Nexus 870 Spectrometer (Thermo Nicolet) equipped with a trapezoid parallelogram ATR geometrymounted on a Harrick Model 4XV 4 × beam condenser in absorption mode (Harrick Scientific, Ossining, NY). The spectrometer was purged with dry and CO_2_-free air supplied by an FT‐IR purge gas generator (Parker Balston, Model 75‐45‐12VDC, Haverhill, MA) for at least one to 3 h before each measurement. An average of 300–1000 scans was used to collect data for each spectrum, and liquid nitrogen-cooled mercury cadmium telluride (MCTA) was utilized as a detector. For easier demonstration, linear baseline corrections were applied using OMNIC software (Baselines Correct) for all spectra.

### SARS-CoV-2 non-structural proteins (NSP) presentation

The target nsp crystallographic structures were obtained from Protein Data Bank (https://www.rcsb.org/), and drawn by PyMOL Molecular Graphics System, Version 1.6^44^.

## Supplementary Information

Supplementary Information.
