# Supplementary material for: Destruction mechanisms of ozone over SARS-CoV-2
Source: Sci Rep. 2021 Sep 22;11:18851. doi: 10.1038/s41598-021-97860-w (PMC8458291; doi:10.1038/s41598-021-97860-w)
Supplement: Supplementary file 1 — Supplementary Information. [file 41598_2021_97860_MOESM1_ESM.pdf]

Supplementary Information

Destruction Mechanisms of Ozone over SARS-CoV-2

Angila Ataei-Pirkooh[1,2], Ali Alavi[3], Mehran Kianirad[4,5], Kowsar Bagherzadeh[6,7], Alireza Ghasempour[8], Omid Pourdakan[8], Reza Adl[10], Seyed Jalal Kiani[1], Mehdi Mirzaei[11], Bita Mehravi[5,8,9*]

[1] Co first Author: Department of Virology, School of Medicine, Iran University of Medical Sciences, Tehran, Iran

[2] Co first Author: Research Center for Clinical Virology, Tehran University of Medical Sciences, Tehran, Iran

[3]Co first Author: Department of Chemistry, Science and Research Branch, Islamic Azad University, Tehran, Iran

[4] Department of Biotechnology, Iranian Research Organization for Science and Technology

[5]Nano Fanavari Kian Gostar Company, Technologies Incubator Center, Iran University of Medical Sciences, Tehran, Iran

[6] Stem Cell and Regenerative Medicine Research Center, Iran University of Medical, Tehran, Iran

[7] Eye Research Center, The Five Senses Institute, Rassoul Akram Hospital, Iran University of Medical Sciences, Tehran, Iran

[8] Department of Medical Nanotechnology, Faculty of Advanced Technologies in Medicine, Iran University of Medical Sciences, Tehran, Iran

[9] Finetech in Medicine Research center; Iran University of Medical, Tehran, Iran

[10] Department of Chemistry, Faculty of Sciences, Shahid Beheshti University, Tehran, Iran

[11]Iran Ministry of Health and Medical Education, Deputy Ministry for Education, Tehran, Iran

***Correspond author: Dr. Bita Mehravi**

Finetech in Medicine Research center, Iran University of Medical, Tehran, Iran
Department of Medical nanotechnology, Faculty of advanced technologies in Medicine, Iran University of Medical Sciences, Tehran, Iran
E-mail: mehravi.b@iums.ac.ir
Tel: +989123840106
Postal Code: 1449614535

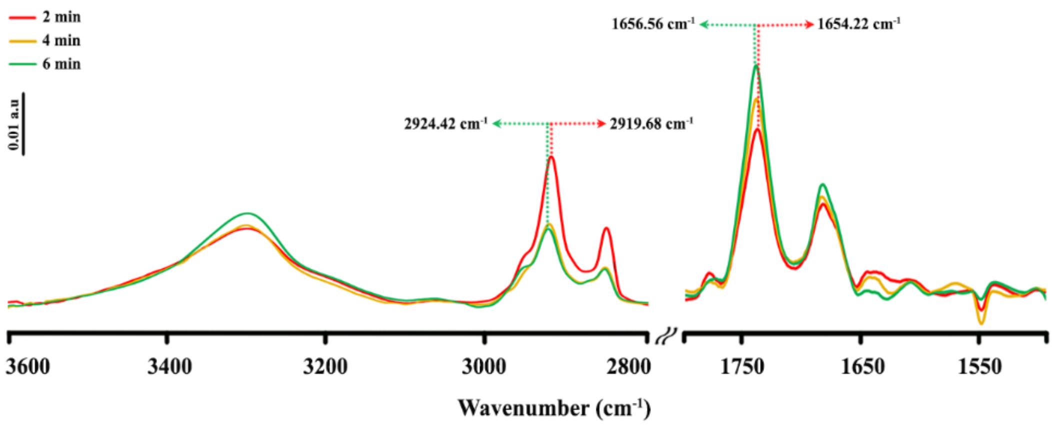

Figure S1. The ATR-FTIR spectra shows the effect of ozone on the virus at different times (2, 4, and 6 minutes). The ATR-FTIR spectra of the virus after 2 minutes(red), 4 minutes(yellow), and 6 minutes (green) of exposure times.

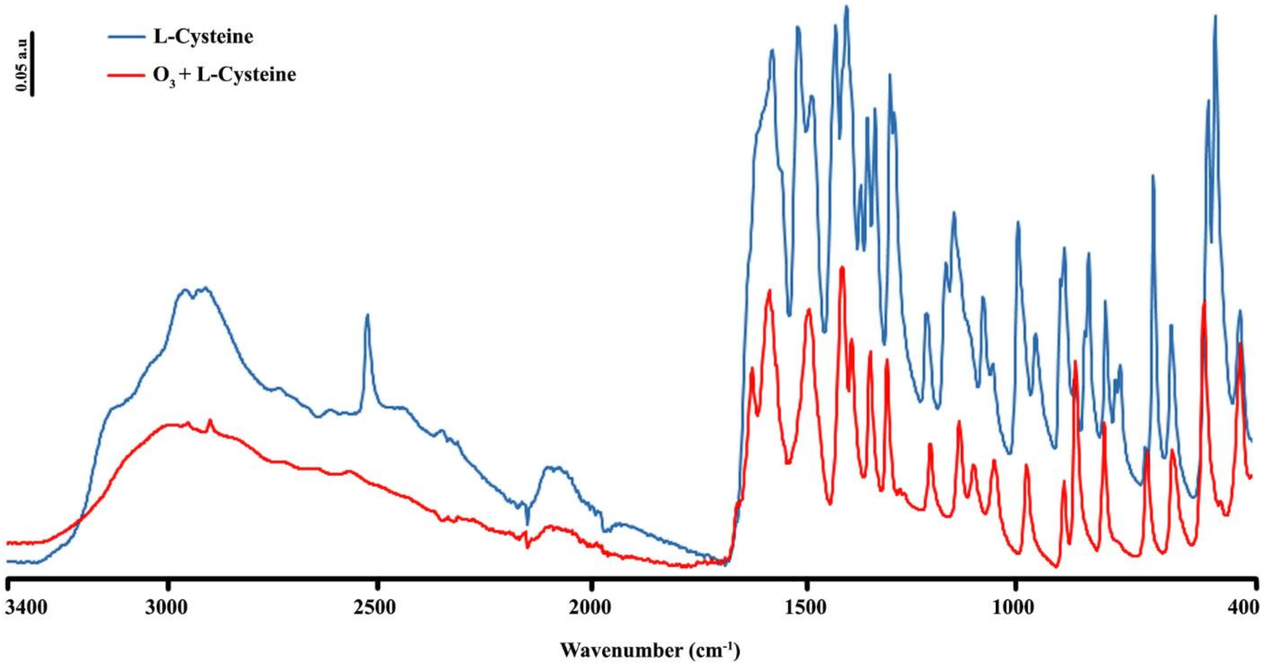

Figure S2. ATR-FTIR spectra to show the conversion of cysteine to cystine.

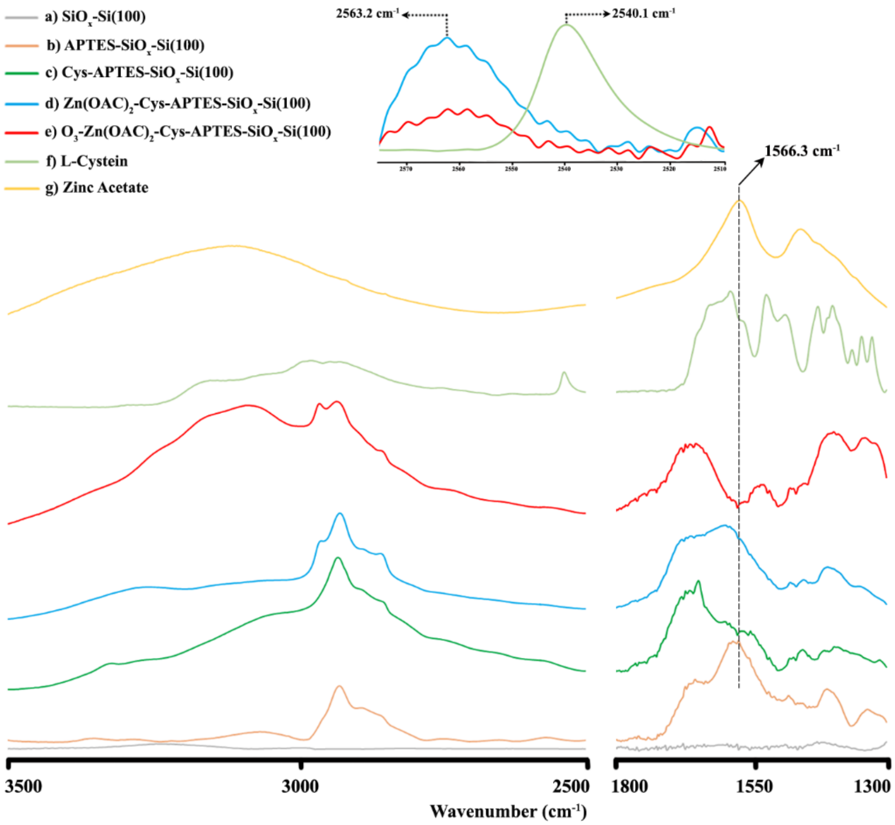

Figure S3. ATR-FTIR spectra to investigate the effect of ozone on Zn bound to cysteine: **a**. IR-Spectra at each modification step, ATR-FTIR spectra of $Zn(OAc)_2$-Cys-APTES-SiO_x Si(100) functionalized surfaces exposed to ozone.

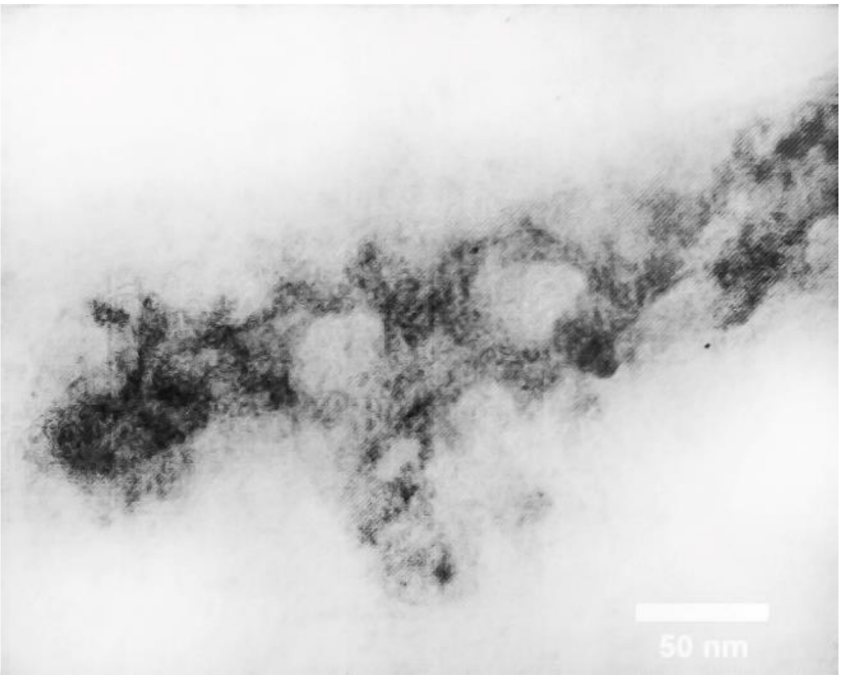

Figure S4. Electron microscopic image of two interconnected viral particles that suggest virus aggregation at 2 minutes of exposure time (77500x).

Table S1. pH decreases in viral suspension with increasing ozone exposure time

**Exposure Time(minutes)**	**pH**
2	6.5
3	5.6
4	5.1
5	4.5
6	4.2
